# ASA^3^P: An automatic and scalable pipeline for the assembly, annotation and higher-level analysis of closely related bacterial isolates

**DOI:** 10.1371/journal.pcbi.1007134

**Published:** 2020-03-05

**Authors:** Oliver Schwengers, Andreas Hoek, Moritz Fritzenwanker, Linda Falgenhauer, Torsten Hain, Trinad Chakraborty, Alexander Goesmann

**Affiliations:** 1 Bioinformatics and Systems Biology, Justus Liebig University Giessen, Giessen, Germany; 2 Institute of Medical Microbiology, Justus Liebig University Giessen, Giessen, Germany; 3 German Center for Infection Research (DZIF), partner site Giessen-Marburg-Langen, Giessen, Germany; Johns Hopkins University, UNITED STATES

## Abstract

Whole genome sequencing of bacteria has become daily routine in many fields. Advances in DNA sequencing technologies and continuously dropping costs have resulted in a tremendous increase in the amounts of available sequence data. However, comprehensive in-depth analysis of the resulting data remains an arduous and time-consuming task. In order to keep pace with these promising but challenging developments and to transform raw data into valuable information, standardized analyses and scalable software tools are needed. Here, we introduce ASA^3^P, a fully automatic, locally executable and scalable assembly, annotation and analysis pipeline for bacterial genomes. The pipeline automatically executes necessary data processing steps, *i*.*e*. quality clipping and assembly of raw sequencing reads, scaffolding of contigs and annotation of the resulting genome sequences. Furthermore, ASA^3^P conducts comprehensive genome characterizations and analyses, *e*.*g*. taxonomic classification, detection of antibiotic resistance genes and identification of virulence factors. All results are presented via an HTML5 user interface providing aggregated information, interactive visualizations and access to intermediate results in standard bioinformatics file formats. We distribute ASA^3^P in two versions: a locally executable Docker container for small-to-medium-scale projects and an OpenStack based cloud computing version able to automatically create and manage self-scaling compute clusters. Thus, automatic and standardized analysis of hundreds of bacterial genomes becomes feasible within hours. The software and further information is available at: asap.computational.bio.

This is a *PLOS Computational Biology* Software paper.

## Introduction

In 1977 DNA sequencing was introduced to the scientific community by Frederick Sanger [1]. Since then, DNA sequencing has come a long way from dideoxy chain termination over high throughput sequencing of millions of short DNA fragments and finally to real-time sequencing of single DNA molecules [2,3]. Latter technologies of so-called next generation sequencing (NGS) and third generation sequencing have caused a massive reduction of time and costs, and thus, led to an explosion of publicly available genomes. In 1995, the first bacterial genomes of *M*. *genitalium* and *H*. *influenzae* were published [4,5]. Today, the NCBI RefSeq database release 93 alone contains 54,854 genomes of distinct bacterial organisms [6]. Due to the maturation of NGS technologies, the laborious task of bacterial whole genome sequencing (WGS) has transformed into plain routine [7] and nowadays, has become feasible within hours [8].

As the sequencing process is not a limiting factor anymore, focus has shifted towards deeper analyses of single genomes and also large cohorts of *e*.*g*. clinical isolates in a comparative way to unravel the plethora of genetic mechanisms driving diversity and genetic landscape of bacterial populations [9]. Comprehensively characterizing bacterial organisms has become a desirable and necessary task in many fields of application including environmental- and medical microbiology [10]. The recent worldwide surge of multi-resistant microorganisms has led to the realization, that without the implementation of adequate measures in 2050 up to 10 million people could die each year due to infections with antimicrobial resistant bacteria alone [11]. Thus, sequencing and timely characterization of large numbers of bacterial genomes is a key element for successful outbreak detection, proper surveillance of emerging pathogens and monitoring the spread of antibiotic resistance genes [12]. Comparative analysis could lead to the identification of novel therapeutic drug targets to prevent the spread of pathogenic and antibiotic-resistant bacteria [13–16].

Another very promising and important field of application for microbial genome sequencing is modern biotechnology. Due to deeper knowledge of the underlying genomic mechanisms, genetic engineering of genes and entire bacterial genomes has become an indispensable tool to transform them into living chemical factories with vast applications, as for instance, production of complex chemicals [17], synthesis of valuable drugs [18–20] and biofuels [21], decontamination and degradation of toxins and wastes [22,23] as well as corrosion protection [24].

Now, that the technological barriers of WGS have fallen, genomics finally transformed into Big Data science [25] inducing new issues and challenges [26]. To keep pace with these developments, we believe that continued efforts are required in terms of the following issues:

a) Automation: Repeated manual analyses are time consuming and error prone. Following the well-known “don’t repeat yourself” mantra and the pareto principle, scientists should be able to concentrate on interesting and promising aspects of data analysis instead of ever repeating data processing tasks.

b) Standard operating procedures (SOPs): In a world of high-throughput data creation and complex combinations of bioinformatic tools SOPs are indispensable to increase and maintain both reproducibility and comparability [27].

c) Scalability: To keep pace with the available data, bioinformatics software needs to take advantage of modern computing technologies, *e*.*g*. multi-threading and cloud computing.

Addressing these issues, several major platforms for the automatic annotation and analysis of prokaryotic genomes have evolved in recent years as for example the NCBI Prokaryotic Genome Annotation Pipeline [6], RAST [28] and PATRIC [29]. All three provide sophisticated genome analysis and annotation pipelines and pose a de-facto community standard in terms of annotation quality. In addition, several offline tools, *e*.*g*. Prokka [30], have been published in order to address major drawbacks of the aforementioned online tools, *i*.*e*. they are not executable on local computers or in on-premises cloud computing environments. However, comprehensive analysis of bacterial WGS data is not limited to the process of annotation alone but also requires sequencing technology-dependent pre-processing of raw data as well as subsequent characterization steps. As analysis of bacterial isolates and cohorts will be a standard method in many fields of application in the near future, demand for sophisticated local assembly, annotation and higher-level analysis pipelines will rise constantly. Furthermore, we believe that the utilization of portable devices for DNA sequencing will shift analysis from central software installations to either decentral offline tools or scalable cloud solutions. To the authors’ best knowledge, there is currently no published bioinformatics software tool successfully addressing all aforementioned issues. In order to overcome this bottleneck, we introduce ASA^3^P, an automatic and scalable software pipeline for the assembly, annotation and higher-level analysis of closely related bacterial isolates.

## Design and implementation

ASA^3^P is implemented as a modular command line tool in Groovy (http://groovy-lang.org), a dynamic scripting language for the Java virtual machine. In order to achieve acceptable to best possible results over a broad range of bacterial genera, sequencing technologies and sequencing depths, ASA^3^P incorporates and takes advantage of published and well performing bioinformatics tools wherever available and applicable in terms of lean and scalable implementation. As the pipeline is also intended to be used as a preprocessing tool for more specialized analyses, it provides no user-adjustable parameters by design and thus facilitates the implementation of robust SOPs. Hence, each utilized tool is parameterized according to community best practices and knowledge (**S1 Table**).

### Workflow, tools and databases

**Fig 1:**
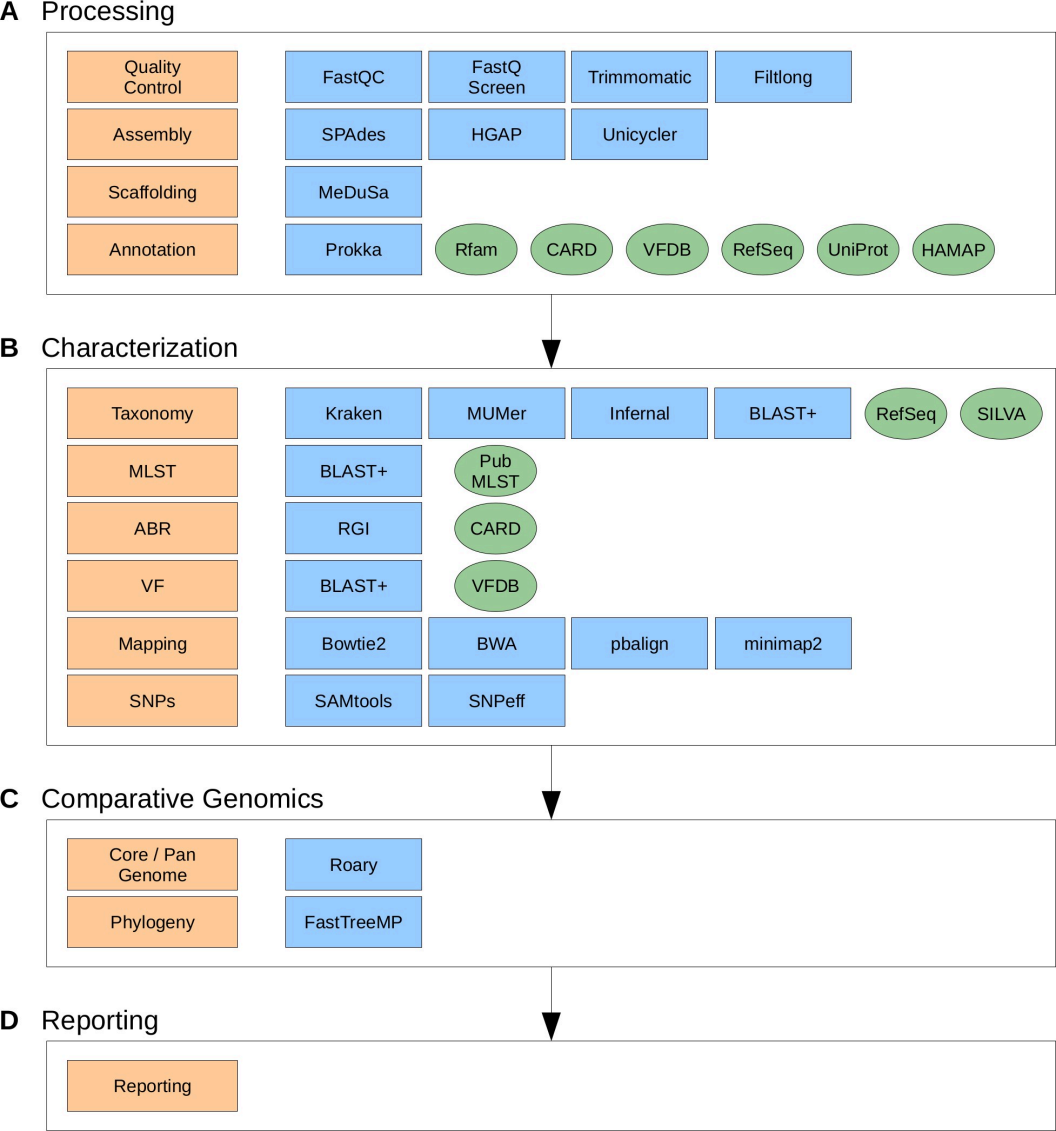
The ASA^3^P workflow and incorporated third party software tools and databases. The ASA^3^P workflow is organized in four stages (large white boxes, A-D) comprising per-isolate processing and characterization, comparative analysis and reporting steps (orange boxes). The processing stage A is mandatory whereas stage B and C are optional and can be skipped by the user. Each step takes advantage of selected third-party software tools (blue boxes) and/or databases (green ovals) depending on the type of provided input data at hand.

Depending on the sequencing technology used to generate the data, ASA^3^P automatically chooses appropriate tools and parameters. An explanation on which tool was chosen for each task is given in **S2 Table**. Semantically, the pipeline’s workflow is divided into four stages (**Fig 1**). In the first mandatory stage A (**Fig 1A**), provided input data are processed, resulting in annotated genomes. Therefore, raw sequencing reads are quality controlled and clipped via FastQC (https://github.com/s-andrews/FastQC), FastQ Screen (https://www.bioinformatics.babraham.ac.uk/projects/fastq_screen), Trimmomatic [31] and Filtlong (https://github.com/rrwick/Filtlong). Filtered reads are then assembled via SPAdes [32] for Illumina reads, HGAP 4 [33] for Pacific Bioscience (PacBio) reads and Unicycler [34] for Oxford Nanopore Technology (ONT) reads, respectively. Hybrid assemblies of Illumina and ONT reads are conducted via Unicycler, as well. Before annotating assembled genomes with Prokka [30], contigs are rearranged and ordered via the multi-reference scaffolder MeDuSa [35]. For the annotation of subsequent pseudogenomes ASA^3^P uses custom genus-specific databases based on binned RefSeq genomes [6] as well as specialized protein databases, *i*.*e*. CARD [36] and VFDB [37]. In order to integrate public or externally analyzed genomes, ASA^3^P is able to incorporate different types of pre-processed data, *e*.*g*. contigs, scaffolds and annotated genomes.

In an optional second stage B (**Fig 1B**), all assembled and annotated genomes are extensively characterized. A taxonomic classification is conducted comprising three distinct methods, *i*.*e*. a kmer profile search, a 16S sequence homology search and a computation of an average nucleotide identity (ANI) [38] against user provided reference genomes. For the kmer profile search, the software takes advantage of the Kraken package [39] and a custom reference genome database based on RefSeq [6]. The 16S based classification is implemented using BLAST+ [40] and the SILVA [41] database. Calculation of ANI values is implemented in Groovy using nucmer within the MUMmer package [42]. A subspecies level multi locus sequence typing (MLST) analysis is implemented in Groovy using BLAST+ [40] and the PubMLST.org [43] database. Detection of antibiotic resistances (ABRs) is conducted via RGI and the CARD [36] database. A detection of virulence factors (VFs) is implemented via BLAST+ [40] and VFDB [37]. Quality clipped reads get mapped onto user provided reference genomes via Bowtie2 [44] for Illumina, pbalign (https://github.com/PacificBiosciences/pbalign) for PacBio and Minimap2 [45] for ONT sequence reads, respectively. Based on these read mappings, the pipeline calls, filters and annotates SNPs via SAMtools [46] and SnpEff [47] and finally computes consensus sequences for each isolate. In order to maximize parallel execution and thus reducing overall runtime, stage A and B are technically implemented as a single step.

A third optional comparative stage C (**Fig 1C**) is triggered as soon as stages A and B are completed, *i*.*e*. all genomes are processed and characterized. Utilizing aforementioned consensus sequences, ASA^3^P computes a phylogenetic approximately maximum-likelihood tree via FastTreeMP [48]. This is complemented by the calculation of a core, accessory and pan-genome as well as the detection of isolate genes conducted via Roary [49].

In a final stage (**Fig 1D**), the pipeline aggregates analysis results and data files and finally provides a graphical user interface (GUI), *i*.*e*. responsive HTML5 documents comprising detailed information via interactive widgets and visualizations. Therefore, ASA^3^P takes advantage of modern web frameworks, *e*.*g*. Bootstrap (https://getbootstrap.com) and jQuery (https://jquery.com) as well as adequate JavaScript visualization libraries, *e*.*g*. Google Charts (https://developers.google.com/chart), D3 (https://d3js.org) and C3 (http://c3js.org).

### User input and output

Each set of bacterial isolates to be analyzed within a single execution is considered as a self-contained analysis of bacterial cohorts and is subsequently referred to as an ASA^3^P project. As ASA^3^P was developed in order to analyze cohorts of closely related isolates, *e*.*g*. a clonal outbreak, the pipeline expects all genomes within a project to belong to at least the same genus, although a common species is most favourable. For each project, the pipeline expects a distinct directory comprising a configuration spreadsheet containing necessary project information and a subdirectory containing all input data files. Such a directory is subsequently referred to as project directory. In order to ease provisioning of necessary information, we provide a configuration spreadsheet template comprising two sheets (**S1 and S2 Figs**). The first sheet contains project meta information such as project names and descriptions as well as contact information on project maintainers and provided reference genomes. The second sheet stores information on each isolate comprising a unique identifier as well as data input type and related files. ASA^3^P is currently able to process input data in the following standard file formats: Illumina paired-end and single-end reads as compressed FastQ files, PacBio RSII and Sequel reads provided either as single unmapped bam files or via triples of bax.h5 files, demultiplexed ONT reads as compressed FastQ files, pre-assembled contigs or pseudogenomes as Fasta files and pre-annotated genomes as Genbank, EMBL or GFF files. In the latter case, corresponding genome sequences can either be included in the GFF file or provided via separate Fasta files.

As ASA^3^P is also intended to be used as an automatic preprocessing tool providing as much reliable information as possible, the results are stored in a standardized manner within project directories comprising quality clipped reads, assemblies, ordered and scaffolded contigs, annotated genomes, mapped reads, detected SNPs as well as ABRs and VFs. In detail, all result files are stored in distinct subdirectories for each analysis by the pipeline and for certain analyses further subdirectories are created therein for each genome (**S3 Fig**). Aggregated information is stored in a standardized but flexible document structure as JSON files. Text and binary result files are stored in standard bioinformatics file formats, *i*.*e*. FastQ, Fasta, BAM, VCF and Newick. Providing results in such a machine-readable manner, ASA^3^P outputs can be further exploited by manual or automatic downstream analyses since customized scripts with a more targeted focus can easily access necessary data. In addition, ASA^3^P creates user-friendly HTML5 reports providing both prepared summaries as well as detailed information via sophisticated interactive visualizations.

### Implementation and software distributions

ASA^3^P is designed as a modular and expandable application with high scalability in mind. It consists of three distinct tiers, *i*.*e*. a command line interface, an application programming interface (API) and analysis specific cluster distributable worker scripts. A common software-wide API is implemented in Java, whereas the core application and worker scripts are implemented in Groovy. In order to overcome common error scenarios on distributed high-performance computing (HPC) clusters and cloud infrastructures and thereby delivering robust runtime behavior, the pipeline takes advantage of a well-designed shared file system-oriented data organization, following a convention over configuration approach. Thus, loosely coupled software parts run both concurrently and independently without interfering with each other. In addition, future enhancements and externally customized scripts reliably find intermediate files at reproducible locations within the file system.

As ASA^3^P requires many third-party dependencies such as software libraries, bioinformatics tools and databases, both distribution and installation is a non-trivial task. In order to reduce the technical complexity as much as possible and to overcome this bottleneck for non-computer-experts, we provide two distinct distributions addressing different use cases and project sizes, *i*.*e*. a locally executable containerized version based on Docker (DV) (https://www.docker.com) as well as an OpenStack (OS) (https://www.openstack.org) based cloud computing version (OSCV). Details and appropriate use cases of both are described in the following sections.

### Docker

For small to medium projects and utmost simplicity we provide a Docker container image encapsulating all technical dependencies such as software libraries and system-wide executables. As the DV offers only vertical scalability, it addresses small projects of less than ca. 200 genomes. The necessary container image is publicly available from our Docker repository (https://hub.docker.com/r/oschwengers/asap) and can be started without any prior installation, except of the Docker software itself. For the sake of lightweight container images and to comply with Docker best practices, all required bioinformatics tools and databases are provided via an additional tarball, subsequently referred to as ASA^3^P volume which users merely need to download and extract, once. For non-Docker savvy users, a shell script hiding all Docker related aspects is also provided. By this, executing the entire pipeline comes down to a single command:

<asap_dir>/asap-docker.sh -p <project_path>.

### Cloud computing

For medium to very large projects, we provide an OS based version in order to utilize horizontal scaling capabilities of modern cloud computing infrastructures. Since creation and configuration of such complex setups require advanced technical knowledge, we provide a shell script taking care of all cloud specific aspects and to orchestrate and execute the underlying workflow logic. Necessary cloud specific properties such as available hardware quotas, virtual machine (VM) flavours and OS identifiers are specified and stored in a custom property file, once. In order to address contemporary demands for high scalability, the OSCV is able to horizontally scale out and distribute workloads on an internally managed Sun Grid Engine (SGE) based compute cluster. A therefore indispensable shared file system is provided by an internal network file system (NFS) server sharing distinct storage volumes for both project data and a necessary ASA^3^P volume. In order to create and orchestrate both software and hardware infrastructures in a fully automatic manner, the pipeline takes advantage of the BiBiGrid (https://github.com/BiBiServ/bibigrid/) framework. Hereby, ASA^3^P is able to adjust the compute cluster size fitting the number of isolates within a project as well as available hardware quotas. Except of an initial VM acting as a gateway into an OS cloud project, the entire compute cluster infrastructure is automatically created, setup, managed and finally shut down by the software. Thus, ASA^3^P can exploit vast hardware capacities and is portable to any OS compatible cloud. For further guidance, all prerequisite installation steps are covered in a detailed user manual.

## Results

### Analysis features

ASA^3^P conducts a comprehensive set of pre-processing tasks and genome analyses. In order to delineate currently implemented analysis features, we created and analyzed a benchmark data set comprising 32 Illumina sequenced *Listeria monocytogenes* isolates randomly selected from SRA as well as four *Listeria monocytogenes* reference genomes from Genbank (**S3 Table**). All isolates were successfully assembled, annotated, deeply characterized and finally included in comparative analyses. **Table 1** provides genome wise minimum and maximum values for key metrics covering results from workflow stages A and B. After conducting a quality control and adapter removal for all raw sequencing reads, a minimum of 393,300 and a maximum of 6,315,924 reads remained, respectively. Genome wise minimum and maximum mean phred scores were 34.7 and 37.2. Assembled genome sizes ranged between 2,818 kbp and 3,201 kbp with a minimum of 12 and a maximum of 108 contigs. Hereby, a maximum N50 of 1,568 kbp was achieved. After rearranging and ordering contigs to aforementioned reference genomes, assemblies were reduced to 2 to 10 scaffolds and 0 to 42 contigs per genome, thus increasing the minimum and maximum N50 to 658 kbp and 3,034 kbp, respectively. Pseudolinked genomes were subsequently annotated resulting in between 2,735 and 3,200 coding genes and between 95 and 144 non-coding genes.

**Table 1:** Common genome analysis key metrics for processing and characterization steps analyzing a benchmark dataset comprising 32 *Listeria monocytogenes* isolates. Minimum and maximum values for selected common genome analysis key metrics resulting from an automatic analysis conducted with ASA^3^P of an exemplary benchmark dataset comprising 32 *Listeria monocytogenes* isolates. Metrics are given for quality control (QC), assembly, scaffolding and annotation processing steps as well as detection of antibiotic resistances and virulence factors characterization steps on a per-isolate level.

Analysis	Metric	Minimum	Maximum
QC	reads	393,300	6,315,924
QC	Mean read length	125.7 nt	228.5 nt
QC	mean Phred score	34.7	37.2
assembly	Genome size	2,817,892 bp	3,201,054 bp
assembly	contigs	12	108
assembly	N50	56,125 bp	1,568,056 bp
assembly	GC content	37%	38%
scaffolding	scaffolds	1	10
scaffolding	contigs	0	42
scaffolding	N50	657,549 bp	3,034,489 bp
annotation	coding genes	2,735	3,200
annotation	non-coding genes	95	144
antibiotic resistance	ABR genes	0	2
virulence factors	VF genes	16	35

After pre-processing, assembling and annotating all isolates, ASA^3^P successfully conducted deep characterizations of all isolates, which were consistently classified to the species level via kmer-lookups as well as 16S ribosomal RNA database searches as *Listeria monocytogenes*, except of a single isolate classified as *Listeria innocua*. In line with these results all isolates shared an ANI value above 95% and a conserved DNA of at least 80% with at least one of the reference genomes, except for the *L*. *innocua* isolate which shared a maximum ANI of 90.7% and a conserved DNA of only 37.3%. Furthermore, the pipeline successfully subtyped all but one of the isolates via MLST, by automatically detecting and applying the “lmonocytogenes” schema. Noteworthy, the *L*. *innocua* isolate constitutes a distinct MLST lineage, *i*.*e*. *L*. *innocua*. ASA^3^P detected between 0 and 2 antibiotic resistance genes and between 16 and 35 virulence factor genes. A comprehensive list of all key metrics for each genome is provided in a separate spreadsheet (**S1 File)**.

Finally, core and pan-genomes were computed resulting in 1,485 core genes and a pan-genome comprising 7,242 genes. Excluding the *L*. *innocua* strain and re-analyzing the dataset reduced the pan-genome to 6,197 genes and increased the amount of core genes to 2,004 additionally endorsing its taxonomic difference.

### Data visualization

**Fig 2:**
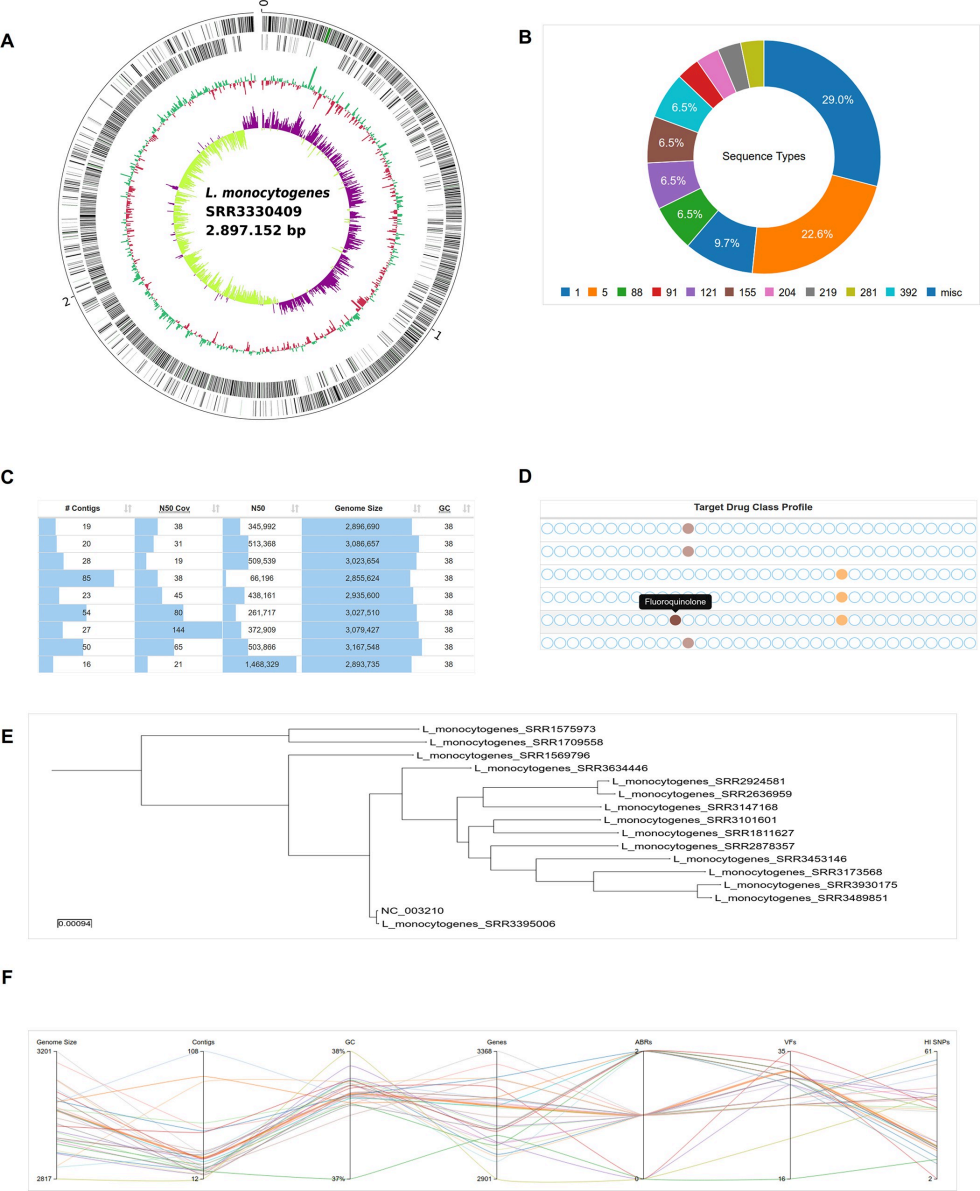
Selection of interactive GUI widgets embedded in generated HTML5 reports. **(A)** Circular genome plot for a *Listeria monocytogenes* pseudogenome. The zoomable and scalable SVG based circular genome plot provides comprehensive information on genome features on mouseover events. Reference-guided rearranged contigs are linked to pseudogenomes for the sake of better readability. From the outermost inward: genes on the forward and reverse strand, respectively, GC content and GC skew. **(B)** Donut chart of MLST sequence type (ST) distribution. The MLST ST distribution of all isolates analyzed within a project is shown by and interactive donut chart. Single STs can be selected or deselected. **(C)** Visual representation of normalized assembly key statistics. Per-isolate assembly key statistics are normalized to minimum and maximum values within a project column-wise and visualized within an interactive data table allowing for column-based sorting and filtering for the rapid comparison of isolates and detection of outliers. **(D)** Antibiotic resistance profile overview widget. An antibiotic resistance profile comprising 34 distinct target drug classes is computed based on CARD annotations for each isolate and transformed into an overview widget allowing a rapid resistome comparison of all analyzed isolates. Black rectangle: a mouseover triggered tooltip describing detected antibiotic target drug resistance. **(E)** SNP-based approximately-maximum-likelihood phylogenetic tree. An approximately-maximum-likelihood phylogenetic tree is computed based on SNPs detected via read-mapping against a reference genome and stored in standard newick file format. The resulting tree is visualized via the interactive Phylocanvas JavaScript library providing comprehensive user interaction features, *e*.*g*. collapsing, expanding and rotating subtrees and tree type selection. **(F)** Parallel coordinates plot providing a multi-dimensional cohort overview of per-isolate genome metrics and characteristics. A selection of seven genome key metrics and characteristics is visualized in a parallel coordinates plot providing a multi-dimensional cohort overview enabling the rapid detection of clustered isolates and outliers. Vertical bars: key metrics or characteristic as plot dimensions; coloured horizontal lines: isolates and related values providing table-synchronized highlighting upon mouseovers.

Analysis results as well as aggregated information get collected, transformed and finally presented by the pipeline via user friendly and detailed reports. These comprise local and responsive HTML5 documents containing interactive JavaScript visualizations facilitating the easy comprehension of the results. Fig 2 shows an exemplary collection of embedded data visualizations. Where appropriate, specialized widgets were implemented, as for instance circular genome annotation plots presenting genome features, GC content and GC skew on separate tracks (**Fig 2A**). These plots can be zoomed, panned and downloaded in SVG format for subsequent re-utilization. Another example is the interactive and dynamic visualization of SNP based phylogenetic trees (**Fig 2E**) via the Phylocanvas library (http://phylocanvas.org) enabling customizations by the user, as for instance changing tree types as well as collapsing and rotating subtrees. In order to provide users with an expeditious but conclusive overview on bacterial cohorts, key genome characteristics are visualized via an interactive parallel coordinates plot (**Fig 2F**) allowing for the combined selection of value ranges in different dimensions. Thus, clusters of isolates sharing high-level genome characteristics can be explored and identified straightforward. In order to rapidly compare different ABR capabilities of individual isolates, a specialized widget was designed and implemented (**Fig 2D**). For each isolate an ABR profile based on detected ABR genes grouped to 34 distinct target drug classes is computed, visualized and stacked for the easy perception of dissimilarities between genomes. Throughout the reports wherever appropriate, numeric results are interactively visualized as, for instance, the distribution of detected MLST sequence types (**Fig 2B**) and per-isolate analysis results summarized via key metrics presented within sortable and filterable data tables (**Fig 2C**).

### Scalability and hardware requirements

When analyzing projects with growing numbers of isolates, local execution can quickly become infeasible. In order to address varying amounts of data, we provide two distinct ASA^3^P distributions based on Docker and cloud computing environments. Each features individual scalability properties and implies different levels of technical complexity in terms of distribution and installation requirements. In order to benchmark the pipeline’s scalability, we measured wall clock runtimes analyzing two projects comprising 32 and 1,024 *L*. *monocytogenes* isolates, respectively (**S3 Table**). Accession numbers for the large data set will be provided upon request. In addition to both public distributions, we also tested a custom installation on an inhouse SGE-based HPC cluster. The DV was executed on a VM providing 32 vCPUs and 64 GB memory. The quotas of the OS cloud project allowed for a total amount of 560 vCPUs and 1,280 GB memory. The HPC cluster comprised 20 machines with 40 cores and 256 GB memory, each. All machines hosted an Ubuntu 16.04 operating system. Table 2 shows the best-of-three runtimes for each version and benchmark data set combination. The pipeline successfully finished all benchmark analyses, except of the 1,024 dataset analyzed by the DV, due to lacking memory capacities required for the calculation of a phylogenetic tree comprising this large amount of genomes. Analyzing the 32 *L*. *monocytogenes* data set on larger compute infrastructures, *i*.*e*. the OS cloud (5:02:24 h) and HPC cluster (4:49:24 h), shows significantly reduced runtimes by approximately 50%, compared to the Docker-based executions (10:59:34 h). Not surprisingly, runtimes of the OSCV are slightly longer than HPC runtimes, due to the inherent overhead of automatic infrastructure setup and management procedures. Excluding these overheads reduces runtimes by approximately half an hour, leading to slightly shorter periods compared to the HPC version. We attribute this to a saturated workload distribution combined with faster CPUs in the cloud as stated in **S4** Table. Comparing measured runtimes for both data sets exhibit a ~5.8- and ~6.9-fold increase for the HPC cluster (27:56:37 h) and OSCV (34:47:45 h) version, respectively, although the amount of isolates was increased 32-fold.

**Table 2:** Wall clock runtimes for each ASA^3^P version utilizing different hardware infrastructures and benchmark dataset sizes. Provided are best-of-three wall clock runtimes for complete ASA^3^P executions analyzing *Listeria monocytogenes* benchmark datasets comprising 32 and 1,024 isolates given in hh:mm:ss format. Docker: a single virtual machine with 32 vCPUs and 64 GB memory was used. Analysis of the 1,024 isolate dataset was not feasible due to memory limitations; HPC: ASA^3^P automatically distributed the workload to an SGE-based high-performance computing cluster comprising 20 nodes providing 40 cores and 256 GB memory each; Cloud: ASA^3^P was executed in an OpenStack based cloud computing project comprising 560 vCPUs and 1,280 GB memory in total. Runtimes in parenthesis exclude build times for automatic infrastructure setups, *i*.*e*. the pure ASA^3^P wall clock runtimes.

	Docker	Cloud	HPC
32 *L*. *monocytogenes*	10:59:34	5:02:24 (4:31:59)	4:49:24
1024 *L*. *monocytogenes*	-	34:47:45 (33:25:26)	27:56:37

We furthermore investigated internal pipeline scaling properties for combinations of fixed and varying HPC cluster and project sizes (**S4 Fig**). In a first setup, growing numbers of *L*. *monocytogenes* isolates were analyzed utilizing a fixed-size HPC cluster of 4 compute nodes providing 32 vCPUs and 64 GB RAM each. Iteratively doubling the amount of isolates from 32 to 1,024 led to runtimes approximately increasing by a factor of 2, in line with our expectations. Nevertheless, we observed an overproportional increase in runtime of the internal comparative steps within stage C compared to the per-isolate steps of stage A and B. We attribute this to the implementations and inherent algorithms of internally used third party executables. As this might become a bottleneck for the analysis of even larger projects, this will be subject to future developments.

In addition, we repetitively analyzed a fixed number of 128 *L*. *monocytogenes* isolates while increasing underlying hardware capacities, *i*.*e*. available HPC compute nodes. In this second setup, we could measure significant runtime reductions for up to 8 compute nodes. Further hardware capacity expansions led to saturated workload distributions and contributed negligible runtime benefits. To summarize all conducted runtime benchmarks, we conclude that ASA^3^P is able to horizontally scale-out to larger infrastructures and thus, conducting expeditious analysis of large projects within favourable periods of time.

To test the reliable distribution and robustness of the pipeline, we executed the DV on an Apple iMac running MacOS 10.14.2 providing 4 cores and 8 GB of memory. ASA^3^P successfully analyzed a downsampled dataset comprising 4 *L*. *monocytogenes* isolates within a measured wall clock runtime of 8:43:12 hours. In order to assess minimal hardware requirements, the downsampled data set was analyzed iteratively reducing provided memory capacities of an OS VM. Hereby, we could determine a minimal memory requirement of 8 GB and thus draw the conclusion that ASA^3^P allows the execution of a sophisticated workflow for the analysis of bacterial WGS data cohorts on ordinary consumer hardware. However, since larger amounts of isolates, more complex genomes or deeper sequencing coverages might result in higher hardware requirements, we nevertheless recommend at least 16 GB of memory.

## Conclusion

We described ASA^3^P, a new software tool for the local, automatic and highly scalable analysis of bacterial WGS data. The pipeline integrates many common analyses in a standardized and community best practices manner and is available for download either as a local command line tool encapsulated and distributed via Docker or a self-orchestrating OS cloud version. To the authors’ best knowledge it is currently the only publicly available tool for the automatic high-throughput analysis of bacterial cohorts WGS data supporting all major contemporary sequencing platforms, offering SOPs, robust scalability as well as a user friendly and interactive graphical user interface whilst still being locally executable and thus offering on-premises analysis for sensitive or even confidential data. So far, ASA^3^P has been used to analyze thousands of bacterial isolates covering a broad range of different taxa.

### Availability and future directions

The source code is available on GitHub under GPL3 license at https://github.com/oschwengers/asap. The Docker container image is accessible at Docker Hub: https://hub.docker.com/r/oschwengers/asap. The ASA^3^P software volume containing third-party executables and databases, OpenStack cloud scripts, a comprehensive manual and configuration templates are hosted at Zenodo: http://doi.org/10.5281/zenodo.3606300. Benchmark and exemplary data projects are hosted sepatately at Zenodo: https://doi.org/10.5281/zenodo.3606761. Questions and issues can be sent to “asap@computational.bio”, bug reports can be filed as GitHub issues.

Albeit ASA^3^P itself is published and distributed under a GPL3 license, some of its dependencies bundled within the ASA^3^P volume are published under different license models, *e*.*g*. CARD and PubMLST. Comprehensive license information on each dependency and database is provided as a DEPENDENCY_LICENSE file within the ASA^3^P directory.

Future directions comprise the development and integration of further analyses, *e*.*g*. detection and characterization of plasmids, phages and CRISPR cassettes as well as further enhancements in terms of scalability and usability.

## Supporting information

S1 Table Third party executable parameters and options. Parameters and options without scientific impact are excluded, *e*.*g*. input/output directories or number of threads.
(PDF)

S2 Table Selection of task-specific third party bioinformatics software tools. Third party bioinformatics software tools selected for each task within ASA^3^P along with a short argumentative reasoning for why they were selected.
(PDF)

S3 Table Accession numbers of 32 *Listeria monocytogenes* isolates and reference genomes of the ASA^3^P benchmark project. This exemplary project comprises 32 isolates from SRR Bioproject PRJNA215355 as well as two *Listeria monocytogenes* reference genomes from RefSeq. The project is provided as a GNU zipped tarball at http://doi.org/10.5281/zenodo.3606761
(PDF)

S4 Table Host CPU information used for wall clock runtime benchmarks. (PDF)

S1 Fig Exemplary screenshot of configuration template sheet 1. (PDF)

S2 Fig Exemplary screenshot of configuration template sheet 2. (PDF)

S3 Fig Exemplary project directory structure. Each project analyzed by ASA^3^P strictly follows a conventional directory organization and thus forestalls the burden of unnecessary configurations. Shown is an exemplary project structure representing input and output files and directories of the *Listeria monocytogenes* example project. For the sake of readability repeated blocks are collapsed represented by a triple dot ‘ …’
(PDF)

S4 Fig Wall clock runtimes for varying compute node and isolate numbers. Runtimes given in hours and separated between comparative and per-isolate internal pipeline stages due to different scalability metrics. Each compute node provides 32 vCPUs and 64 GB memory. *L*. *monocytogenes* strains were randomly chosen from SRA Bioproject PRJNA215355. (**A)** Runtimes of a fixed-size compute cluster comprising 4 compute nodes analyzing varying isolate numbers. (**B)** Runtimes of compute clusters with varying numbers of compute nodes analyzing a fixed amount of 128 isolates.
(PDF)

S1 File Comprehensive list of all per-genome key metrics. (XLS)
