# Supplementary material for: ASA3P: An automatic and scalable pipeline for the assembly, annotation and higher-level analysis of closely related bacterial isolates
Source: PLoS Comput Biol. 2020 Mar 5;16(3):e1007134. doi: 10.1371/journal.pcbi.1007134 (PMC7077848; doi:10.1371/journal.pcbi.1007134)
Supplement: S1 Table — Parameters and options without scientific impact are excluded, e.g. input/output directories or number of threads. (PDF) [file pcbi.1007134.s001.pdf]

S1 Table. Third party executable parameters and options. Parameters and options without scientific impact are excluded, *e.g.* input/output directories or number of threads.

Tool	Parameters
Trimmomatic	“ILLUMINACLIP:...:2:30:10”<br>“LEADING:15”<br>“TRAILING:15”<br>“SLIDINGWINDOW:4:20”<br>“MINLEN:20”<br>“TOPHRED33”
Filtlong	--min_length 500<br>--min_mean_q 85<br>--min_window_q 65
FastQ Screen	--aligner bowtie2' (bwa for PacBio)<br>--subset 1000 (for PacBio)
SPAdes	--careful<br>--disable-gzip-output<br>--cov-cutoff auto<br>--phred-offset 33
HGAP	Pbalign.task_options.min_accuracy: 70<br>Pbalign.task_options.no_split_subreads: false<br>Genomic_consensus.task_options.min_confidence: 40<br>falcon_ns.task_options.HGAP_GenomeLength_str: 6000000<br>Pbcoretools.task_options.read_length: 0<br>Genomic_consensus.task_options.use_score: 0<br>Pbalign.task_options.min_length: 50<br>Pbalign.task_options.algorithm_options: --minMatch 12 --bestn 10 --minPctSimilarity 70.0<br>Pbalign.task_options.hit_policy: randombest<br>Pbcoretools.task_options.other_filters: rq >= 0.7<br>Pbalign.task_options.concordant: false<br>Genomic_consensus.task_options.min_coverage: 5<br>falcon_ns.task_options.HGAP_SeedCoverage_str: 30<br>falcon_ns.task_options.HGAP_AggressiveAsm_bool: false<br>Genomic_consensus.task_options.algorithm: best<br>falcon_ns.task_options.HGAP_SeedLengthCutoff_str: -1<br>Genomic_consensus.task_options.diploid: false
MeDuSa	-random 100
Prokka	--usegenus<br>--force<br>--addgenes<br>--rfam<br>--rawproduct
cmsearch (taxonomy, 16S)	--rfam<br>--noali
blastn (taxonomy, 16S)	-evalue 1E-10
blastn (MLST)	-ungapped

<table>
<tr><td></td><td>-dust no
-evalue 1E-20
-word_size 32
-culling_limit 2
-perc_identity 95</td></tr>
<tr><td>blastp (VF)</td><td>-culling_limit 2</td></tr>
<tr><td>RGI (ABR)</td><td>--input_type contig</td></tr>
<tr><td>bowtie2 (mapping)</td><td>--sensitive</td></tr>
<tr><td>minimap2 (mapping)</td><td>-a
-x map-ont</td></tr>
<tr><td>samtools mpileup (SNP detection)</td><td>-uRI</td></tr>
<tr><td>bcftools call (SNP detection)</td><td>--variants-only
--skip-variants indels
--output-type v
--ploidy 1
-c</td></tr>
<tr><td>SNPsift filter (SNP detection)</td><td>"(QUAL >= 30) & ((na FILTER) | (FILTER = 'PASS')) & (DP >= 20) & (MQ >= 20)"</td></tr>
<tr><td>SNPeff ann (SNP detection)</td><td>-nodownload
-no-intron
-no-downstream
-no SPLICE_SITE_REGION
-upDownStreamLen 250</td></tr>
<tr><td>bcftools consensus (phylogenetic tree)</td><td>--haplotype 1</td></tr>
<tr><td>fasttreemp</td><td>-nt
-boot 100</td></tr>
<tr><td>roary</td><td>-e
-n
-cd 100
-g 100000</td></tr>
</table>
