# Supplementary material for: ASA3P: An automatic and scalable pipeline for the assembly, annotation and higher-level analysis of closely related bacterial isolates
Source: PLoS Comput Biol. 2020 Mar 5;16(3):e1007134. doi: 10.1371/journal.pcbi.1007134 (PMC7077848; doi:10.1371/journal.pcbi.1007134)
Supplement: S2 Table — Third party bioinformatics software tools selected for each task within ASA3P along with a short argumentative reasoning for why they were selected. (PDF) [file pcbi.1007134.s002.pdf]

S2 Table. Selection of task-specific third party bioinformatics software tools. Third party bioinformatics software tools selected for each task within ASA³P along with a short argumentative reasoning for why was selected.

Task - Tool	Parameters
QC - Trimmomatic	- Published<br>- Well performing (due to publication)<br>- Community standards and best practices
QC - FiltLong	- Well performing (broad experience)<br>- One of the first tools available, broadly used
QC - FastQC	- Well performing (broad experience)<br>- Community standards and best practices<br>- Broad applicability (all sequencing platforms)<br>- Actively maintained
QC - FastQ Screen	- Well performing (broad experience)<br>- Broad applicability (all sequencing platforms)<br>- Actively maintained
Assembly Illumina - SPAdes	- Well performing (publication & broad experience)<br>- Community standards and best practices<br>- Actively maintained
Assembly PacBio - HGAP	- Well performing (publication & broad experience)<br>- One of the first tools available<br>- Actively maintained
Assembly NanoPore/Hybrid (Illumina) - Unicycler	- Well performing (publication & broad experience)<br>- Unicycler combines trimming, polishing and dnaA rotation like no other assembly pipeline whilst still being easy to technically integrate
Scaffolding - MeDuSa	- Well performing (publication & broad experience)<br>- Supporting multiple references<br>- In contradiction to many other multi-reference scaffolders, MeDuSa is available as a locally executable tool
Annotation - Prokka	- Well performing (publication & broad experience)<br>- Community standards and best practices<br>- Actively maintained
ABR - CARD rgi	- Well designed AMR ontology<br>- All-in-one AMR detection tool (acquired genes, mutation based, efflux pump mediated)<br>- Actively maintained
Pan/Core Genome calculation - Roary	- Well performing (publication & broad experience)<br>- Community standards and best practices<br>- Computationally applicable for large cohorts<br>- Actively maintained

Phylogenomics - FastTree	- Well performing (publication & broad experience)<br>- Computationally applicable for large cohorts<br>- Community standards and best practices
