# Supplementary material for: ASA3P: An automatic and scalable pipeline for the assembly, annotation and higher-level analysis of closely related bacterial isolates
Source: PLoS Comput Biol. 2020 Mar 5;16(3):e1007134. doi: 10.1371/journal.pcbi.1007134 (PMC7077848; doi:10.1371/journal.pcbi.1007134)
Supplement: S3 Table — This exemplary project comprises 32 isolates from SRR Bioproject PRJNA215355 as well as two Listeria monocytogenes reference genomes from RefSeq. The project is provided as a GNU zipped tarball at http://doi.org/10.5281/zenodo.3606761 (PDF) [file pcbi.1007134.s003.pdf]

S3 Table. Accession numbers of 32 *Listeria monocytogenes* isolates and reference genomes of the ASA³P benchmark project. This exemplary project comprises 32 isolates from SRR Bioproject PRJNA215355 as well as two *Listeria monocytogenes* reference genomes from RefSeq. The project is provided as a GNU zipped tarball at http://doi.org/10.5281/zenodo.3606761

Type	Accession numbers
reference	NC_003210.1<br>NC_022568.1<br>NZ_CP019164.1<br>NZ_CP019615.1
isolates	SRR3330409, SRR1810516, SRR2924581, SRR3101601, SRR3634446, SRR3181835, SRR2982078, SRR3574517, SRR1575973, SRR3930175, SRR1973978, SRR2140707, SRR2976738, SRR2636959, SRR3173568, SRR3928673, SRR1709558, SRR1514752, SRR3489851, SRR1811627, SRR2878357, SRR1272887, SRR3147168, SRR2533768, SRR1569796, SRR1763858, SRR3395006, SRR3930198, SRR2861532, SRR2562281, SRR3453146, SRR3137565
