# Supplementary material for: ASA3P: An automatic and scalable pipeline for the assembly, annotation and higher-level analysis of closely related bacterial isolates
Source: PLoS Comput Biol. 2020 Mar 5;16(3):e1007134. doi: 10.1371/journal.pcbi.1007134 (PMC7077848; doi:10.1371/journal.pcbi.1007134)
Supplement: S4 Table — (PDF) [file pcbi.1007134.s004.pdf]

S4 Table. Host CPU information used for wall clock runtime benchmarks.

Infrastructure	Host CPU
Docker OS cloud VM	- Intel(R) Xeon(R) CPU E5-2690 v4 @ 2.60GHz<br>- 2x 14 cores without hyperthreading
OS cloud	- Intel(R) Xeon(R) CPU E5-2690 v4 @ 2.60GHz<br>- 2x 14 cores without hyperthreading
HPC cluster	- Intel(R) Xeon(R) CPU E5-2670 v2 @ 2.50GHz<br>- 2x 10 cores with hyperthreading
