# Supplementary material for: ASA3P: An automatic and scalable pipeline for the assembly, annotation and higher-level analysis of closely related bacterial isolates
Source: PLoS Comput Biol. 2020 Mar 5;16(3):e1007134. doi: 10.1371/journal.pcbi.1007134 (PMC7077848; doi:10.1371/journal.pcbi.1007134)
Supplement: S1 Fig — (PDF) [file pcbi.1007134.s005.pdf]

Name	Fda-lmonocytogenes
Description	A subset of 32 clinical/environmental Listeria monocytogenes isolates…
Genus	Listeria

User

Name	Oliver
Surname	Schwengers
Email	oliver.schwengers@computational.bio.uni-giessen.de

Reference Genomes

Reference Genome List	NC_003210-Listeria-monocytogenes-EGDe.gbk
	NC_022568-Listeria-monocytogenes-EGD.gbk
	NZ_CP019164-Listeria-monocytogenes-strain-HPB2088.gbk
	NZ CP019615-Listeria-monocytogenes-strain-10-092876-0168.gbk

S1 Fig. Exemplary screenshot of configuration template sheet 1.
