# Supplementary material for: ASA3P: An automatic and scalable pipeline for the assembly, annotation and higher-level analysis of closely related bacterial isolates
Source: PLoS Comput Biol. 2020 Mar 5;16(3):e1007134. doi: 10.1371/journal.pcbi.1007134 (PMC7077848; doi:10.1371/journal.pcbi.1007134)
Supplement: S2 Fig — (PDF) [file pcbi.1007134.s006.pdf]

Species	Strain	Input	File 1	[File 2]	[File 3]
monocytogenes	SRR3330409	paired-end	SRR3330409_1.fastq.gz	SRR3330409_2.fastq.gz	
monocytogenes	SRR1810516	paired-end	SRR1810516_1.fastq.gz	SRR1810516_2.fastq.gz	
monocytogenes	SRR2924581	paired-end	SRR2924581_1.fastq.gz	SRR2924581_2.fastq.gz	
monocytogenes	SRR3101601	[illegible]	SRR3101601_1.fastq.gz	SRR3101601_2.fastq.gz	
monocytogenes	SRR3634446	[illegible]	SRR3634446_1.fastq.gz	SRR3634446_2.fastq.gz	
monocytogenes	SRR3181835	[illegible]	SRR3181835_1.fastq.gz	SRR3181835_2.fastq.gz	
monocytogenes	SRR2982078	[illegible]	SRR2982078_1.fastq.gz	SRR2982078_2.fastq.gz	
monocytogenes	SRR3574517	[illegible]	SRR3574517_1.fastq.gz	SRR3574517_2.fastq.gz	
monocytogenes	SRR1575973	[illegible]	SRR1575973_1.fastq.gz	SRR1575973_2.fastq.gz	
monocytogenes	SRR3930175	[illegible]	SRR3930175_1.fastq.gz	SRR3930175_2.fastq.gz	
monocytogenes	SRR1973978	paired-end	SRR1973978_1.fastq.gz	SRR1973978_2.fastq.gz	

single / paired-end / mate-pairs / pacbio-rs2 / pacbio-sequel / nanopore / nanopore-pe / contigs / contigs-ordered / genome

S2 Fig. Exemplary screenshot of configuration template sheet 2.
