# Supplementary figures and images for: ASA3P: An automatic and scalable pipeline for the assembly, annotation and higher-level analysis of closely related bacterial isolates
Source: PLoS Comput Biol. 2020 Mar 5;16(3):e1007134. doi: 10.1371/journal.pcbi.1007134 (PMC7077848; doi:10.1371/journal.pcbi.1007134)

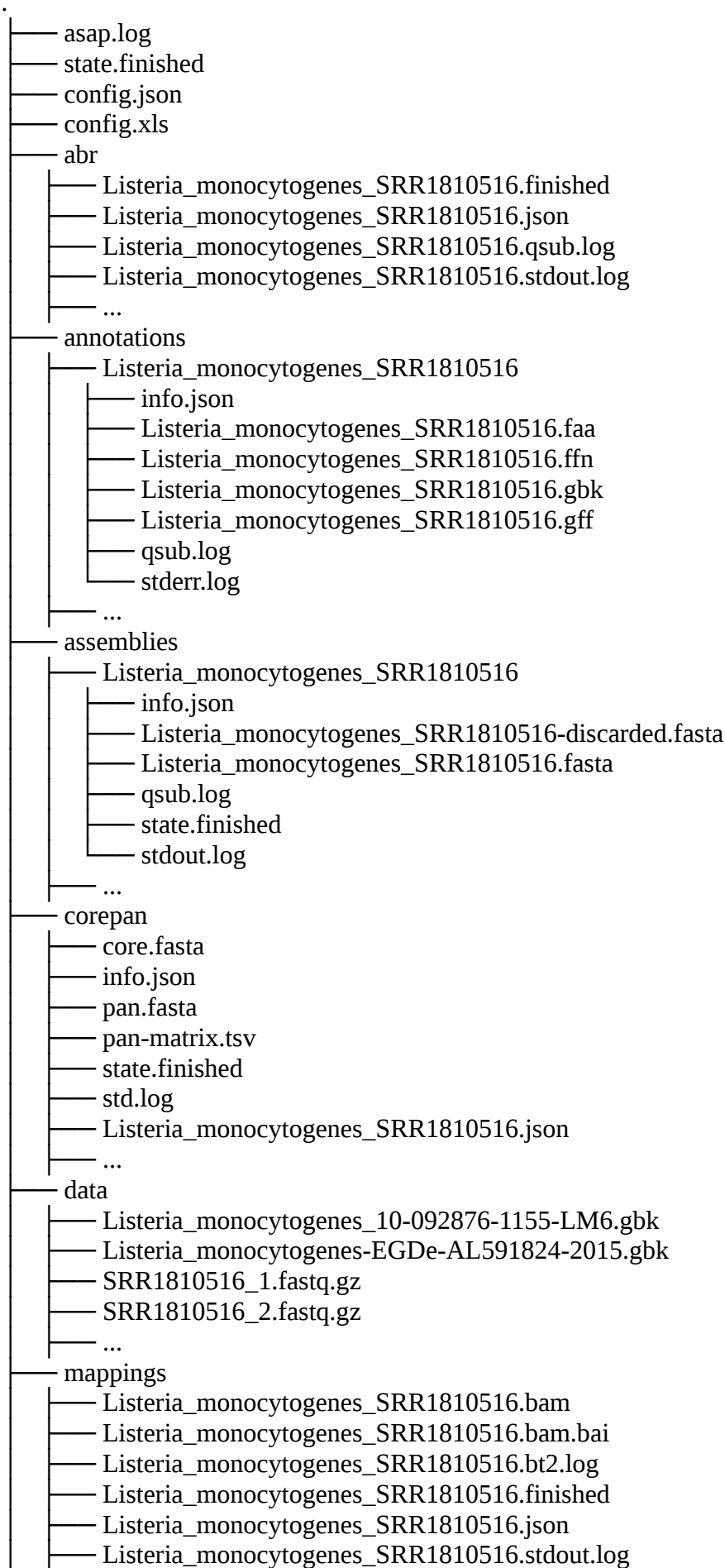

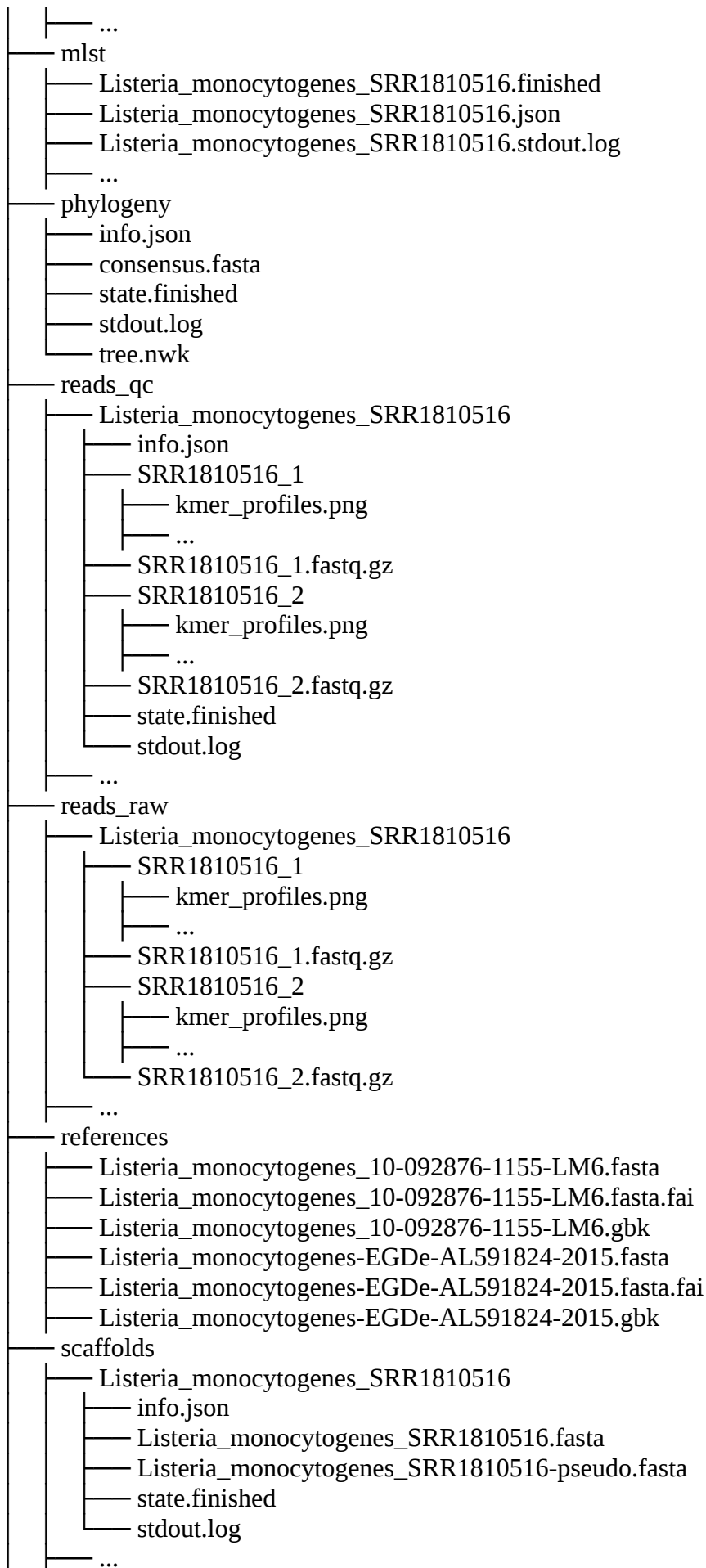

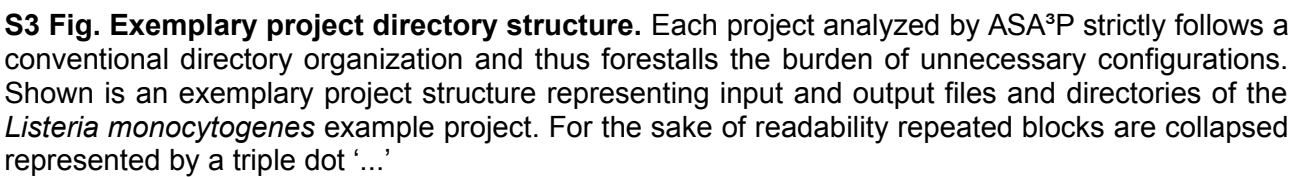

Supplement: S3 Fig — Each project analyzed by ASA3P strictly follows a conventional directory organization and thus forestalls the burden of unnecessary configurations. Shown is an exemplary project structure representing input and output files and directories of the Listeria monocytogenes example project. For the sake of readability repeated blocks are collapsed represented by a triple dot ‘ …’ (PDF) [file pcbi.1007134.s007.pdf]
