# Supplementary material for: ASA3P: An automatic and scalable pipeline for the assembly, annotation and higher-level analysis of closely related bacterial isolates
Source: PLoS Comput Biol. 2020 Mar 5;16(3):e1007134. doi: 10.1371/journal.pcbi.1007134 (PMC7077848; doi:10.1371/journal.pcbi.1007134)
Supplement: S4 Fig — Runtimes given in hours and separated between comparative and per-isolate internal pipeline stages due to different scalability metrics. Each compute node provides 32 vCPUs and 64 GB memory. L. monocytogenes strains were randomly chosen from SRA Bioproject PRJNA215355. (A) Runtimes of a fixed-size compute cluster comprising 4 compute nodes analyzing varying isolate numbers. (B) Runtimes of compute clusters with varying numbers of compute nodes analyzing a fixed amount of 128 isolates. (PDF) [file pcbi.1007134.s008.pdf]

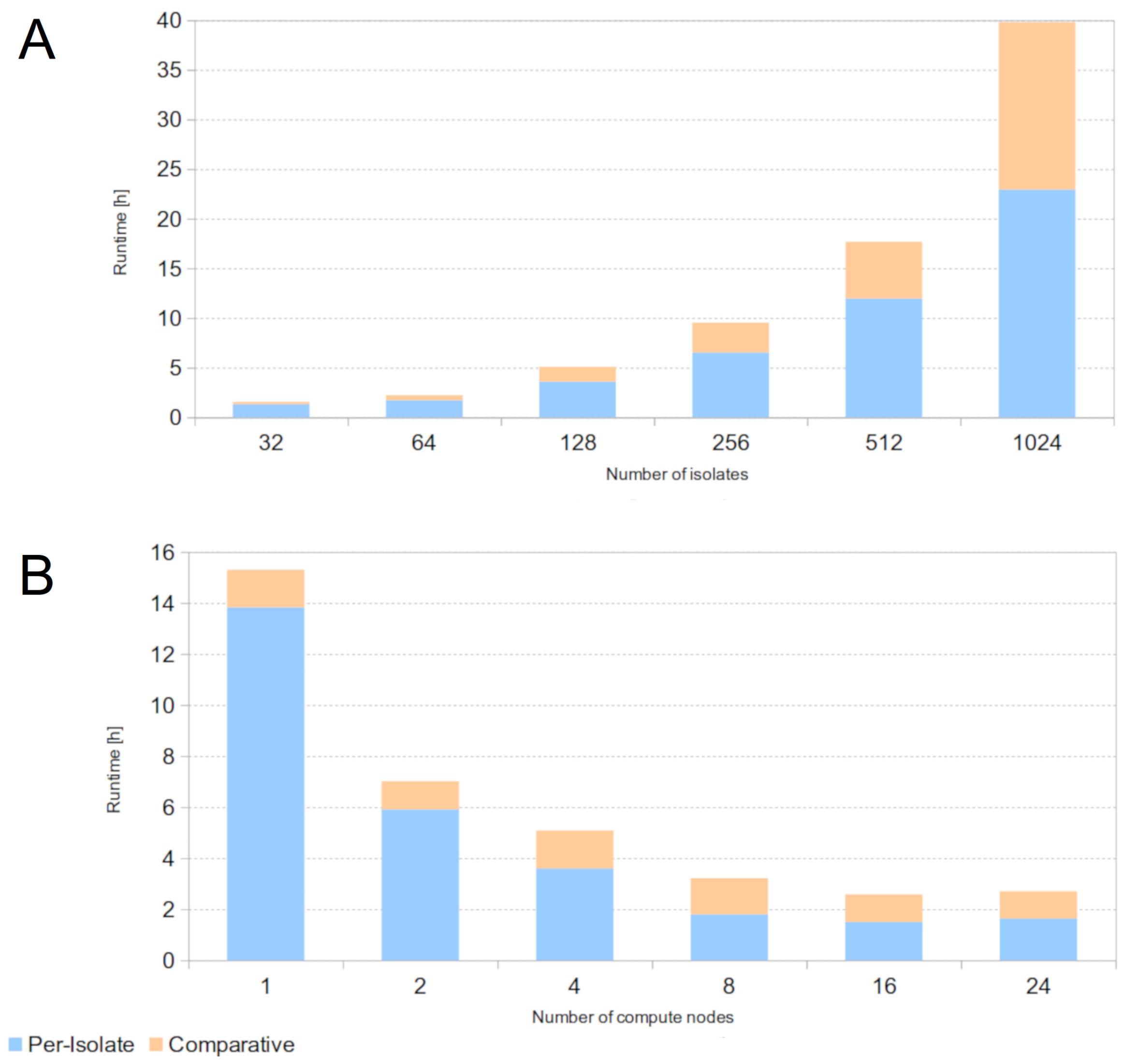

S4 Fig. Wall clock runtimes for varying compute node and isolate numbers. Runtimes given in hours and separated between comparative and per-isolate internal pipeline stages due to different scalability metrics. Each compute node provides 32 vCPUs and 64 GB memory. *L. monocytogenes* strains were randomly chosen from SRA Bioproject PRJNA215355. (A**)** Runtimes of a fixed-size compute cluster comprising 4 compute nodes analyzing varying isolate numbers. (B**)** Runtimes of compute clusters with varying numbers of compute nodes analyzing a fixed amount of 128 isolates.
